# Epithelial stem cells as mucosal antigen-delivering cells: a novel preventive approach

**DOI:** 10.1186/1471-2334-14-S2-O4

**Published:** 2014-05-23

**Authors:** Marie-Claire Gauduin, Robert White, Nicole Chenciner, Kathleen Vincent, Patrice Frost, Philippe Blancou

**Affiliations:** 1 Texas Biomedical Research Institute, San Antonio, Texas, USA

## Introduction

HIV transmission occurs predominantly across mucosal surfaces. An ideal preventive strategy would be to target HIV at mucosal entry sites to prevent infection. We developed a novel epithelial stem cells-based AIDS preventive approach in female macaques. This approach is based on the ability of therapeutic lentiviral vectors integrated in mucosal epithelial stem cells to induce virus-specific cellular immune responses at mucosal sites of viral entry. We first intended to expose the cervicovaginal tract to conditions used for intravaginal vaccine delivery and non-invasively image local tissues to determine the vaccination effects on epithelial integrity and generation of antigen-specific mucosal immune responses.

## Methods

Experimental cervicovaginal vaccinations were carried out on 12 uninfected female macaques, in conjunction with deliberate abrasion of epithelium using Depo-Provera, 4% nonoxynol-9 treatments, or gentle abrasions to reduce vaginal epithelial height and cell layers number. Longitudinal study was performed for baseline levels of hormones, antibodies, cytokine profiles during normal cycling with/without vaccination. Endoscopic colposcopy followed by optical coherence tomography (OCT) helped monitor macaque cervical-vaginal epithelium pre-, post-, without treatment to measure epithelial thickness changes. Colposcopy help visualizing the vagina and cervix. Blood samples and biopsies were collected at various time-points to evaluate immune responses to vaccination.

## Results

OCT imaging provided quantitative measurements of epithelial changes and detected minute changes in epithelial thickness and morphology. Colposcopy and OCT imaging correlated with hormone levels and biopsies imaging. Depo-Provera gave better results in epithelium thinning but naturally cycling macaques showed clearer thickness differences. All collected secretions, biopsies and blood samples gave a better understanding of the correlation between menstrual cycle, hormonal level, epithelial thickness changes. Optimal conditions for vaccine delivery were determined and revealed virus-specific T cell response in all vaccinated animals.

## Conclusions

OCT is well suited for the examination of superficial mucosal layers and close underlying stromal structures of tissues. In this study, we demonstrated the feasibility of using epithelial stem cells as mucosal antigen-presenting cells to induce viral specific immune responses at mucosal sites, and improved our knowledge of the role of mucosal surfaces.

